# Supplementary material for: The interactome of intact mitochondria by cross-linking mass spectrometry provides evidence for coexisting respiratory supercomplexes
Source: Mol Cell Proteomics. 2017 Dec 8;17(2):216–32. doi: 10.1074/mcp.RA117.000470 (PMC5795388; doi:10.1074/mcp.RA117.000470)
Supplement: Supplemental Data [file supp_RA117.000470_133922_0_supp_23978_fzffwf.zip › spectra_annotation/mito_DR_spectra_annotation/52-1-5-1-23-1.pdf]

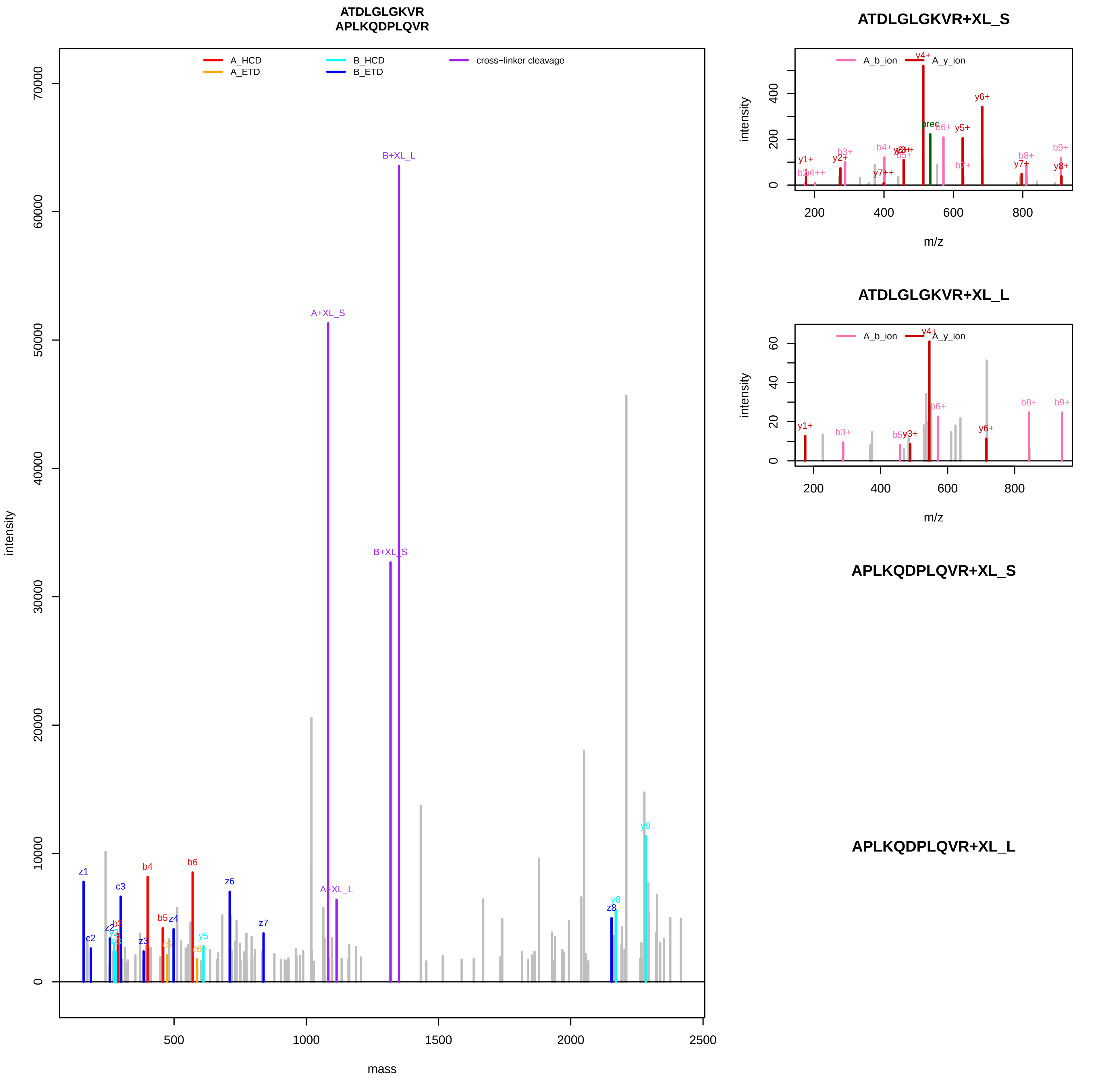
ATDLGLGKVR
APLKQDPLQVR
A_HCD
A_ETD
B_HCD
B_ETD
cross−linker cleavage
intensity
mass
B+XL_L
A+XL_S
B+XL_S
A+XL_L
z1
c2
z2
b3
c3
b4
z3
b5
z4
b6
c5
c6
y5
z6
z7
z8
y8
y9
ATDLGLGKVR+XL_S
A_b_ion
A_y_ion
y4+
y6+
prec
b6+
y5+
b4+
y3+
b5+
b3+
y2+
y1+
b8+
b9+
y7+
y8+
b7+
y7++
m/z
ATDLGLGKVR+XL_L
y6+
b3+
b5+
APLKQDPLQVR+XL_S
APLKQDPLQVR+XL_L
